# Signs and symptoms of pediatric complex regional pain syndrome - type 1: A retrospective cohort study

**DOI:** 10.1080/24740527.2023.2179917

**Published:** 2023-04-18

**Authors:** Giulia Mesaroli, Logan McLennan, Yvonne Friedrich, Jennifer Stinson, Navil Sethna, Deirdre Logan

**Affiliations:** a Department of Physical Therapy, University of Toronto, Toronto, Ontario, Canada; b Department of Rehabilitation, The Hospital for Sick Children, Toronto, Ontario, Canada; c Child Health Evaluative Sciences, Research Institute, The Hospital for Sick Children, Toronto, Ontario Canada; d Department of Child Study and Human Development, Tufts University, Medford, Massachusetts, USA; e Department of Clinical Psychology, Friedrich-Schiller University Jena, Jena, Germany; f Lawrence S. Bloomberg Faculty of Nursing, University of Toronto, Toronto, Ontario, Canada; g Department of Anesthesiology, Critical Care and Pain Medicine, Boston Children’s Hospital, Boston, Massachusetts, USA; h Department of Anaesthesia, Harvard Medical School, Boston, Massachusetts, USA; i Department of Psychiatry, Harvard Medical School, Boston, Massachusetts, USA

**Keywords:** complex regional pain syndrome, pediatric pain, symptoms

## Abstract

**Background:**

Complex regional pain syndrome (CRPS) presents with an array of symptoms that can vary from child to child, making it difficult to diagnose and differentiate from other pain conditions such as chronic musculoskeletal (MSK) pain. Thirteen symptoms and signs are outlined in the Budapest criteria for CRPS (developed and validated for adults) but have not been well described in pediatrics.

**Aims:**

The aim of this study was to describe the signs and symptoms of pediatric CRPS type 1 (CRPS 1) and determine whether a cluster of symptoms can differentiate CRPS 1 from chronic MSK pain.

**Methods:**

A retrospective cohort study of pediatric patients with CRPS 1 and MSK pain in a pediatric pain program was conducted. Descriptive statistics were used to report demographics and pain characteristics. The chi-square test was used to evaluate differences in signs and symptoms between patients with CRPS and MSK pain. A logistic regression model was used to evaluate whether a cluster of symptoms could predict a diagnosis of CRPS 1.

**Results:**

The sample included 187 patients (99 with CRPS 1 and 88 with MSK pain); 81% were female with a mean age 14.1 years. The most prevalent CRPS symptoms were hyperalgesia (54%) and allodynia (52%). A cluster of symptoms (hyperalgesia, color changes, and range of motion) predicted the probability of a diagnosis of CRPS 1.

**Conclusions:**

A cluster of symptoms may be critical in differentiating pediatric CRPS 1 and MSK pain. Future research is needed to determine if this model is valid in external populations and to explore whether a similar model can differentiate CRPS 1 from other pain conditions (e.g., neuropathic pain).

## RÉSUMÉ

**Contexte:** Le syndrome douloureux régional complexe présente un éventail de symptômes qui peuvent varier d’un enfant à l’autre, ce qui rend difficile le diagnostic et la différenciation des autres affections douloureuses telles que la douleur musculo-squelettique chronique. Treize symptômes et signes sont décrits dans les critères de Budapest pour le syndrome douloureux régional complexe (développés et validés pour les adultes) mais ceux-ci n’ont pas encore été bien décrits en pédiatrie.

**Objectifs:** L’objectif de cette étude était de décrire les signes et symptômes du syndrome douloureux régional complexe pédiatrique de type 1 et de déterminer si un groupe de symptômes pouvait le différencier de la douleur musculo-squelettique chronique.

**Méthodes:** Une étude de cohorte rétrospective de patients pédiatriques atteints de syndrome douloureux régional complexe de type 1 et de douleur musculo-squelettique chronique dans le cadre d’un programme de lutte contre la douleur pédiatrique a été mis en place. Des statistiques descriptives ont été utilisées pour rendre compte des données démographiques et des caractéristiques de la douleur. Le test du chi carré a été utilisé pour évaluer les différences dans les signes et les symptômes entre les patients souffrant de syndrome douloureux régional complexe et de douleur musculo-squelettique chronique. Un modèle de régression logistique a été utilisé pour évaluer si un groupe de symptômes pourrait prédire un diagnostic de syndrome douloureux régional complexe de type 1 en pédiatrie.

**Résultats:** L’échantillon comprenait 187 patients pédiatriques (99 atteints de syndrome douloureux régional complexe de type 1 et 88 atteints de douleur musculo-squelettique chronique); 81 % étaient de sexe féminin, avec un âge moyen de 14,1 ans. Les symptômes de syndrome douloureux régional complexe de type 1 pédiatrique les plus fréquents étaient l’hyperalgésie (54 %) et l’allodynie (52 %). Un groupe de symptômes (hyperalgésie, changements de couleur et amplitude de mouvement) prédisait la probabilité d’un diagnostic de syndrome douloureux régional complexe d type 1 pédiatrique.

**Conclusions:** Un groupe de symptômes peut être essentiel pour différencier le syndrome douloureux régional complexe de type 1 pédiatrique de la douleur musculo-squelettique chronique. D’autres études sont nécessaires pour déterminer si ce modèle est valide parmi les populations externes et pour déterminer si un modèle similaire peut différencier le syndrome douloureux régional complexe de type 1 pédiatrique d’autres conditions douloureuses (p. ex., la douleur neuropathique).

## Introduction

In children, complex regional pain syndrome (CRPS) is a rare but debilitating disease. Although the pathophysiology is not fully understood, a number of factors have been proposed including neurogenic inflammation, maladaptive plasticity, and sensitization of nociceptors.^1^ In pediatrics, more than 95% of cases are CRPS type 1 (CRPS 1; in the absence of neurological damage), the peak incidence is among early adolescent females who most commonly experience pain in the lower extremity.^2^ This differs from CRPS in adults, with a peak incidence in females age 50 to 59 and predominance in the upper extremity.^3–5^ CRPS can result in severe pain-related disability including avoidance of social environments, absence from school, physical impairments, and disorders of mood or sleep.^6,7^ To mitigate disability, emotional distress, and financial burden, early diagnosis and intervention are critical; the International Association for the Study of Pain specifically recommends a 1-week wait time for chronic pain clinics for patients in the acute phase of CRPS.^8,9^

Screening and diagnosis in the context of pediatric CRPS is challenging and relies on clinical judgment and expertise. At present, there are no valid diagnostic or screening tools for CRPS in children, although several diagnostic tools exist for adults. Determining a clinical diagnosis can be difficult. Although CRPS has hallmark symptoms (e.g., allodynia, edema, color changes), there are many potential symptoms, and the combination of symptoms can vary from child to child. Additionally, some of these symptoms are sometimes seen in other pain conditions, such as chronic musculoskeletal (MSK) pain persistent for ≥3 months, making it challenging to differentiate between the two conditions.^10–13^

Thirteen symptoms and signs have been outlined in the Budapest criteria for CRPS.^14^ Budapest criteria were developed based on data obtained from examination of adults with CRPS.^15^ The signs and symptoms detailed in the Budapest criteria are divided into four categories based on statistical analysis performed during validation studies in adults and do not necessarily represent physiological relatedness.^14^ Further research is needed to investigate the intrinsic connection among these 13 signs and symptoms to elucidate the possible pathology in the pediatric population. This is critical to advance our understanding of the content validity of diagnostic and screening tools in pediatric CRPS, including currently available tools developed for adults (e.g., Budapest criteria, Valencia criteria) and tools being developed for pediatrics (e.g., Pediatric PainSCAN). Valencia criteria are an updated version of the Budapest criteria, where the sign and symptom criteria are mirrored with additional criteria to evaluate the impact on emotions and functioning and reduce diagnostic ambiguity.^16^ Pediatric PainSCAN is a screening tool for pediatric neuropathic pain and CRPS currently under development.^17^ A key indicator of content validity includes the relevance of the items in diagnostic or screening tool.^18^ Understanding which symptoms of pediatric CRPS, corresponding to items in these tools, are most prevalent and most discriminant in making a diagnosis would inform the validity of available tools and aid in developing or revising tools.

The objectives of this study were to (1) describe the signs and symptoms of CRPS 1 in the pediatric population and (2) explore whether a cluster of symptoms can differentiate CRPS 1 from chronic MSK pain.

## Materials and Methods

### Setting

Data were collected from patients in a tertiary care pediatric pain rehabilitation center (PPRC). The data were collected as part of a registry (approved by the Boston Children's Hospital Internal Review Board #IRB-P00015943) in which participants provided written informed consent for future use of their data.

### Dataset

The PPRC is day treatment facility for children and adolescents up to 21 years of age with chronic pain conditions (e.g., MSK pain, CRPS). The data set included 262 participants with a diagnosis of either CRPS 1 or MSK pain admitted to the PPRC between 2009 to 2017; complete data on the presence of signs and symptoms were collected for a subset of randomly selected participants (*n* = 187). Data were extracted from the patient’s electronic health record by a research assistant using a data extraction spreadsheet. Data were collected on patient demographics, pain characteristics, and 13 signs and 13 symptoms of CRPS based on the Budapest criteria (Table 1). Pain characteristics and signs and symptom data were collected from the physician’s note from the patient’s initial intake visit.

**Table 1.** Budapest clinical criteria.^19^

1	Continuing pain that is disproportionate to any inciting event
2	Must report at least one symptom in *three of the four* following categories.
3	Must display at least one sign at the time of evaluation in *two or more* of the following categories.
4	There is no other diagnosis that better explains the signs and symptoms.
	**Categories**
	Sensory: evidence of hyperalgesia (to pinprick) and/or allodynia (to light touch and/or deep somatic pressure and/or joint movement)
	Vasomotor: evidence of temperature asymmetry and/or skin color changes and/or asymmetry
	Sudomotor/edema: evidence of edema and/or sweating changes and/or sweating asymmetry
	Motor/trophic: evidence of decreased range of motion and/or motor dysfunction (weakness, tremor, dystonia) and/or trophic changes (hair, nails, skin)

### Variables

Demographic variables (age, sex, and race) were extracted from the demographic profile of patients’ electronic health records. Race was dichotomized to White vs non-White due to small numbers. Pain characteristic variables included pain location, duration, spread, intensity, and symptom severity. Pain duration was defined as the number of months since the onset of pain, as self-reported by the patient during the initial intake visit. Symptom severity was defined as the number of symptoms present (range 0–13).

Each sign and symptom were categorized as present or absent. These included allodynia, hyperalgesia, color changes, edema, temperature changes, weakness, reduced range of motion, tremors, dystonia, sweating changes, hair growth, nail growth, and skin changes. With respect to signs, the following methods and equipment were used in the assessment: (1) allodynia to light touch: stroking the skin with a cotton-woven sterile gauze sponge over a 3–5 cm distance; (2) allodynia to temperature: copper rod placed on the skin (copper rod was rubbed a few times with a cotton-woven sterile gauze sponge to raise the temperature of the rod); (3) hyperalgesia: pain with pin prick to the skin using a 25-gauge needle loaded on a 3 cc syringe to move freely; (4) temperature difference >1°C: noncontact infrared digital thermometer.^20–22^ The remaining signs were assessed through observation. The outcome for the secondary objective was patient diagnosis, either CRPS 1 or MSK pain. These were clinical diagnoses made by a physician with pediatric pain expertise during the initial intake visit.

### Statistical Analysis

Descriptive statistics (frequencies, means, medians) were used to describe patient demographic characteristics and pain characteristics. For bivariate analyses, a chi-square test was used to compare frequency distributions between categorical variables, and Fisher’s exact test was used when the total number of observations in any category was less than five. For continuous variables, independent samples *t* test was used for parametric data, and Mann-Whitney *U* test was used for nonparametric data.

To address the secondary objective and explore whether a cluster of symptoms can differentiate a diagnosis of CRPS 1 from chronic MSK pain, a prediction model using logistic regression was used. Variable selection methods to determine which of the 13 symptoms to include in the model are described. First, one variable (weakness) was not selected for inclusion based on clinical rationale. Weakness is present in most chronic pain conditions and therefore unlikely to predict any specific diagnosis. Second, variables were then screened for missing data, low cell counts (less than ten), and statistical significance with between-group analyses; variables with a *P* value > 0.25 were not selected. No variables were eliminated based on significant missing data (>10%). The symptoms sweating and dystonia were not selected for the model because there were cell counts less than ten in one category of the variable and the difference in these variables between groups was not statistically significant (*P* > 0.25). Third, the all-possible regressions method was used to examine regression models with the remaining ten variables to identify a model that provides the highest (or near highest) *R*^2^ with the minimum number of variables. Given that the results of this analysis will help inform screening and diagnostic tools for CRPS 1, the least number of symptoms was considered desirable to minimize the number of questions/items in self-report tools.

The omnibus likelihood ratio test was used to determine whether any variables in the final model were significant (*P* < 0.05 was considered indication of good fit). The significance of individual variables in the model was tested using the Wald test. Additional model assumptions were examined including (1) model fit tested with the Hosmer-Lemeshow (*P* > 0.05 indicates good fit) and C-statistic (higher values indicating better fit), (2) the model is not overspecified/overfitted, (3) influential observations (tested using casewise diagnostics with DFBeta), and (4) multicollinearity of variables (symptoms with tolerance <0.25 was considered collinear; clinical judgment was used to determine which symptoms would remain if found to be collinear). A model validation approach (such as split sample) was not completed because it was not computationally possible due to the small sample size.

Statistical analysis was performed using SAS On Demand for Academics.^23^ A two-tailed *P* value <0.05 was considered statistically significant.

## Results

### Primary Objective

There were 187 patients in this data set, including 99 patients with CRPS 1 and 88 with MSK pain. Overall, there were little/no missing data in the variables included in the data set (<1%). Patient demographic and pain characteristics are presented in Table 2 for the entire sample (*n* = 187). In the entire sample, 81% were female (*n* = 153) with a mean age 14.1 years. There was a statistically significant difference (*P* = 0.034) in age between patients with CRPS 1 (mean 13.7 years) and MSK pain (mean 14.5 years); all other demographic variables were consistent between groups. With respect to pain characteristics, there were significant differences (*P* < 0.05) between groups with respect to pain duration, pain location, and pain spread: patients with CRPS 1 had shorter pain duration (median 8 months vs. 12.5 months), greater proportion of lower extremity (82% vs. 32%), and unilateral presentation (87% vs. 33%) when compared to patients with MSK pain.

**Table 2.** Patient characteristics stratified by diagnosis (*N* = 187).

Characteristic	CRPS (*n* = 99)	MSK (*n* = 88)	*P* value
Sex, *n* (%)			
Female	83 (83.8)	70 (79.9)	0.45^a^
Male	16 (16.2)	18 (20.1)	
Age (years), mean ± SD	13.65 ± 2.66	14.48 ± 2.64	0.034*^c^
Race, *n* (%)			
White	93 (94.9)	76 (86.4)	0.11^a^
Non-white	5 (5.1)	10 (11.5)	
Unknown	0 (0.0)	2 (2.2)	
Pain duration (months), median (IQR)	8 (4, 14)	12.5 (7.5, 36.0)	<0.001*^b^
Pain intensity (NRS), median (IQR)	7 (6, 8)	7 (5, 8)	0.069^b^
Constant pain, *n* (%)			
Yes	99 (100.0)	85 (96.6)	0.102^a^
No	0 (0.0)	3 (3.4)	
Pain location, *n* (%)			
Upper extremity	15 (15.2)	6 (6.8)	<0.001*^a^
Lower extremity	81 (81.8)	34 (38.6)	
Trunk	2 (2.0)	28 (31.8)	
Face/head	0 (0.0)	3 (3.4)	
Diffuse	1 (1.0)	17 (19.3)	
Length of stay (days), median (IQR)	4 (4, 5)	4 (3, 4)	0.013*^b^
Pain spread, *n* (%)			
Unilateral	86 (86.9)	29 (33.0)	<0.001*^a^
Bilateral	10 (10.1)	11 (12.5)	
Central	2 (2.0)	30 (34.1)	
Diffuse	1 (1.0)	18 (20.5)	
Symptom severity (0–13), median (IQR)	4 (3, 6)	1 (0, 3)	<0.001*^b^
Budapest clinical criteria met, *n* (%)			
Yes	63 (63.6)	10 (11.4)	<0.001*^a^
No	36 (36.4)	78 (88.6)	

*Statistically significant; a = chi-square; b = Wilcoxon, c = *T* test.

IQR = interquartile range, NRS = numeric rating scale (0–10).

The prevalence of symptoms and signs is presented in Table 3. In patients with CRPS 1, the most prevalent symptoms were hyperalgesia (73%), allodynia (67%), color changes (64%), and weakness (48%). The most prevalent signs were hyperalgesia (75%), allodynia (70%), temperature changes (68%), and weakness (54%). Few patients with CRPS (<5%) presented with sweating changes or tremors.

**Table 3.** Patient symptoms and signs stratified by diagnosis (*N* = 187).

	CRPS (*n* = 99)	MSK (*n* = 88)	*P* value
Symptoms, *n* (%) present			
Hyperalgesia	**73 (73.7)**	***28 (31.8)***	<0.0001*
Allodynia	**66 (66.7)**	***32 (36.6)***	0.0001*
Temperature changes	***32 (32.3)***	9 (10.2)	0.0003*
Color changes	**63 (63.6)**	14 (15.9)	<0.0001*
Edema	***44 (44.4)***	12 (13.6)	<0.0001*
Sweating changes	5 (5.0)	2 (2.3)	0.45^a^
Decreased range of motion	***43 (43.4)***	14 (15.9)	<0.0001*
Weakness	***47 (47.5)***	***31 (35.2)***	0.090
Tremor	11 (11.1)	5 (5.7)	0.18
Dystonia	5 (5.0)	2 (2.3)	0.45^a^
Hair changes	15 (15.2)	0 (0.0)	<0.0001*^a^
Nail changes	21 (21.2)	5 (5.7)	0.002*
Skin changes	13 (13.1)	3 (3.4)	0.019*^a^
Signs, *n* (%) present			
Hyperalgesia	**74 (74.8)**	19 (21.6)	<0.0001*
Allodynia	**69 (69.7)**	***34 (38.7)***	<0.0001*
Temperature changes	***32 (32.3)***	5 (5.7)	<0.0001*
Color changes	***42 (42.9)***	6 (6.8)	<0.0001*
Edema	28 (28.3)	4 (4.6)	<0.0001*^a^
Sweating changes	4 (4.0)	0 (0.0)	0.12^a^
Decreased range of motion	***44 (44.4)***	17 (19.3)	0.0003*
Weakness	***53 (53.5)***	23 (26.1)	0.0001*
Tremor	1 (1.0)	2 (2.3)	0.60^a^
Dystonia	11 (11.1)	3 (3.4)	0.054
Hair changes	14 (7.5)	0 (0.0)	<0.0001*^a^
Nail changes	13 (13.1)	0 (0.0)	0.0002*^a^
Skin changes	13 (13.1)	2 (2.3)	0.0066*^a^

*Statistically significant; a = Fisher’s exact test (all others used chi-Square); **Bold **= >60% present, ***italics*** = 30%–60% present. Symptoms were self-reported by the patient, whereas signs were observed on clinical examination.

With respect to differences in the presence of signs and symptoms between patients with CRPS 1 and MSK pain, the presence of 10 of 13 signs and 9 of 13 symptoms was significantly different between groups. The signs and symptoms sweating, tremor, and dystonia did not significantly differ between groups. Differences in the presence of signs and symptoms was also analyzed in the subset of patients with extremity pain (*n* = 136) and are presented in Supplemental Table 1. Sixty-four percent of patients with CRPS 1 (*n* = 63) met the Budapest clinical criteria, resulting in a sensitivity of 64% and specificity of 89%.

### Secondary Objective

In total, 187 patients were included in the logistic regression model exploring symptoms predictive of a diagnosis of CRPS 1. The final set of symptoms for inclusion was selected based on the all-possible regression method. This method indicated that a three-variable model had the near-highest *R*^2^ (62) value (hyperalgesia, color changes, and decreased range of motion). Models with additional symptoms resulted in only marginal increases in the *R*^2^ (up to 67 with all 10 symptoms). A model with four symptoms (adding the symptom hair growth changes) was explored (*R*^2^ = 65), but this resulted in overspecification of the model due to too few patients with the symptom present. Results of the final model are presented in Table 4. The omnibus likelihood ratio test was significant (*P* < 0.0001), indicating that at least one variable in the model was significant. Each individual variable in the model was significant (*P* < 0.05) according to the Wald test. This cluster of symptoms (hyperalgesia, color changes, and reduced range of motion) predicted the probability of a diagnosis of CRPS 1 in comparison to MSK pain, whereby the presence of these symptoms increased the odds of a diagnosis of CRPS 1. The presence of the symptom color changes resulted in the highest increase in odds of a diagnosis of CRPS 1 (adjusted odds ratio = 6.62; 95% confidence interval 3.10, 14.15).

**Table 4.** Multivariable logistic regression analysis of symptoms predictive of CRPS (*N* = 187).

Variable	Adjusted odds ratio (95% confidence interval)	Test statistic	*P* value
Hyperalgesia	3.40 (1.66, 6.94)	11.2	0.008
Color changes	6.62 (3.10, 14.15)	23.8	<0.0001
Decreased range of motion	3.37 (1.51, 7.53)	8.8	0.003

Reference for all symptoms was present vs. absent. Omnibus likelihood ratio: chi-square test statistic (degrees of freedom), *P* value: 70.7(3), <0.0001.

The model appeared to have good fit. The Hosmer-Lemeshow goodness of fit test suggested that the model does fit the data (*P = *0.8). The C-statistic indicates that the model has good discrimination (0.83). No variables in the model were considered collinear (tolerance values for each symptom were >0.8) Upon visual inspection using casewise diagnostics with DFBeta, few influential outliers were noted. These few outliers were considered negligible and were not removed from the analyses. There were no indications of overspecification of the model.

## Discussion

This study describes the presence of signs and symptoms of CRPS 1 in a cohort of children in a tertiary care pediatric pain program. Significant differences were noted in the presence of signs and symptoms in children diagnosed with CRPS 1 as opposed to children diagnosed with chronic MSK pain. Our results also indicate that a presence of a cluster of symptoms can differentiate a diagnosis of CRPS 1 (hyperalgesia, color changes, and reduced range of motion) from chronic MSK pain.

In this study, the most prevalent symptoms in children with CRPS 1 were sensory changes and color changes, and the most prevalent signs were sensory changes and weakness. Our results align with existing studies of signs and symptoms of CRPS in Canadian youth by Mesaroli et al.^6^ (*n* = 59) and Baerg et al.^2^ (*n* = 168). Across all three studies (including this study), the most prevalent symptoms included allodynia (67%–84%) and color changes (64%–87%) and most prevalence signs included allodynia (70%–82%) and weakness (54%–76%). Color changes were a more prevalent symptom (64%–87%) than sign (40%–66%) in each study. Trophic changes (hair, nail, and skin changes) were present in less than 25% of cases probably due to early diagnosis, and treatment and movement disorders (tremors, dystonia) were present in less than 20% cases across all three studies.^2,6^

Differences in the prevalence rates of symptoms versus signs may be related several factors. First, clinical features of CRPS are episodic in nature, and patients may be able to self-report symptoms experienced in the prior days that are not present during the time of the physical examination. Perhaps some clinical features such as color changes, swelling, and temperature are more episodic—that is, dynamic regional autonomic nervous system dysregulation due to immobilization of the limb—than others (e.g., skin texture, nail and hair growth changes) due to chronic poor circulation to deliver nutrition over time, explaining this difference in prevalence rates across symptoms vs. signs. Second, patients may be able to report symptoms that have resolved and are not present on physical exam. Third, some symptoms may be difficult for patients to report because of persistent pain and immobilization of the affected limb (e.g., sweating changes, temperature changes, weakness) that may be more easily detected as a sign on physical exam using objective measures (e.g., skin thermometer, sweat test, manual muscle testing).

### Differences in Pediatric vs Adult CRPS

Our results highlight some similarities and differences in clinical features of CRPS between adults and children. Our results suggest that some symptoms are relatively rare in children (e.g., trophic changes, tremors, and dystonia). A large cohort study by Ott and Maihoefner^5^ examined signs and symptoms of more than 1000 participants (primarily adults) with CRPS. In this cohort, the mean age was 50 years, >70% of cases presented in the upper extremity, and mean disease duration was 37 ± 78 weeks. In comparison to our study, this study reported higher prevalence rates of trophic changes (47% vs. 13%), tremors (31% vs. 1%), and sweating changes (43% vs. 4%). There are a number of possible reasons to explain these differences. Firstly, in our study, 82% of cases presented in the lower extremity, and the location of the disease may contribute to physiological differences in symptom presentation related to the anatomy or function of the limb. Secondly, although the mean disease duration was similar, the standard deviation was much wider in the study by Ott and Maihoefner,^5^ indicating disease chronicity spanned up to 2 years in adults, which is longer than that in our study in pediatrics (approximately 1 year). Longer disease duration can lead to worsening of disease-related disability in adults, including more disuse symptoms, and could explain the higher prevalence rates of symptoms.

### Implications for Diagnostic and Screening Tools

Our study indicates poor sensitivity (64%) but good specificity (89%) of the Budapest criteria in the pediatric population when using a clinical diagnosis by a pediatric pain physician as a reference test. Generally, a minimum sensitivity of 70% is considered acceptable for screening and diagnostic tools.^24,25^ According to a 2020 systematic review, there are no other existing studies examining the validity of the Budapest criteria in pediatrics.^15^ The Budapest criteria in adults display significantly higher rates of sensitivity (up to 99%) and similar rates of specificity.^15^ This disagreement between the Budapest criteria and physician diagnosis in our study may be in part due to a cutoff score that is too high for pediatrics. Some autonomic system symptoms and signs are relatively rare due to varying responses of maturing autonomic system from birth to late adolescence.^26^ Additionally, the Budapest criteria do not adequately capture signs that are episodic or resolve over time, yet these nuances in clinical features may weigh into a physician’s judgment when making a clinical diagnosis. As such, pediatric patients may not meet the threshold for a positive result on the Budapest criteria. In clinical practice and future research, caution should be taken if applying the Budapest criteria (or the updated Valencia criteria) in pediatric populations. With respect to screening tools, our results indicate that a cluster of symptoms can predict the probability of a diagnosis of CRPS 1 in children. The Pediatric PainSCAN is a screening tool for pediatric neuropathic pain and CRPS currently under development; our results suggest that these symptoms (hyperalgesia, color changes, reduced range of motion) are critical to include as screening questions in the final version of the tool.

### Significance for Future Research

Pediatric chronic pain conditions can sometimes present with overlapping symptoms, and in the absence of well-validated diagnostic tests, it can be difficult to distinguish between subtypes.^27^ Overlap between symptoms and co-existence of multiple chronic pain conditions may be related to shared physiological mechanisms, including central sensitization.^28,29^ However, clinical expertise and some research point to clusters of symptoms that are distinct between chronic pain conditions, such as CRPS, neuropathic pain, and fibromyalgia.^30–32^ Differentiating between types of chronic pain conditions has important implications for the future of pain research and ultimately clinical practice. Future research aiming to evaluate interventions and determine incidence/prevalence rates, prognosis, phenotypes, and genetic etiologies would benefit from validated tools to define study populations and achieve more homogenous samples. For example, randomized controlled trials of pediatric chronic pain would benefit from evaluating interventions accounting for potential key factors that might influence treatment response. These factors include not only the type of chronic pain but also age, sex, pain phenotype, and co-existing or past history of other chronic pain diagnoses.

### Limitations

Our study is limited in that the sample size was small with respect to the number of patients with CRPS 1 (*n* = 99) and overall sample size (*N* = 187). First, the small sample size impacted our ability to validate our exploratory model (e.g., using a split sample approach) of symptoms predictive of CRPS. This may result in a model that is not generalizable to other settings. Second, due to sample size limitations, our exploratory model was specific to symptoms (not signs) and did not include other factors that might be able to differentiate CRPS from chronic MSK pain (e.g., pain location). Third, although the data were extracted from the physician’s initial assessment, this was not necessarily at the early onset of disease due to delays in referral or time spent on the clinic wait list. As such, patients may have presented with varying symptom severity depending on the stage of disease at the initial physician's assessment (acute, chronic, recovery or relapse). Fourth, participants in this sample were predominantly White, and our results may not be generalizable to pediatric patients of other races with CRPS. This data set spans a period of 9 years (2009–2017), which may include refinements in approach to CRPS assessment over time. However, given that the Budapest criteria were first introduced in 2007 and only recently updated in 2021, changes in assessment approach were minimal and unlikely to impact our results.^16,19^

## Conclusion

Although there are many potential signs and symptoms of CRPS, some symptoms (e.g., sensory changes, weakness, and color changes) were much more prevalent than others (e.g., sweating changes, tremors, nail changes) in our pediatric cohort. In differentiating children with CRPS and MSK pain, a cluster of symptoms present (hyperalgesia, color changes, and decreased range of motion) predicted the probability of a diagnosis of CRPS 1. Future research is needed to standardize objective assessments, including psychophysical testing, to determine whether a cluster of symptoms can predict a diagnosis of CRPS 1 in relation to other differential chronic pain diagnoses (e.g., other neuropathic pain conditions).

## Supplementary Material

Supplemental Material
