# Supplementary material for: Signs and symptoms of pediatric complex regional pain syndrome - type 1: A retrospective cohort study
Source: Can J Pain. 2023 Apr 18;7(1):2179917. doi: 10.1080/24740527.2023.2179917 (PMC10116922; doi:10.1080/24740527.2023.2179917)
Supplement: Supplemental Material [file UCJP_A_2179917_SM3296.docx]

**Supplemental Table. Patient symptoms and signs stratified by diagnosis in subset of patients with extremity pain (*n* = 136)**

|  | **CRPS**  **(*n*=96)** | **MSK**  **(*n*=40)** | ***p* value** |
| --- | --- | --- | --- |
| **Symptoms – n (%) present** | | | |
| Hyperalgesia | **70 (72.9%)** | ***15 (37.5%)*** | 0.0001* |
| Allodynia | **64 (66.7%)** | ***19 (47.5%)*** | 0.037* |
| Temperature changes | ***32 (33.3%)*** | 7 (17.5%) | 0.063 |
| Color changes | **63 (65.6%)** | 10 (25.0%) | <0.001* |
| Edema | ***44 (45.8%)*** | 7 (17.5%) | 0.002* |
| Sweating changes | 5 (5.2%) | 2 (5.0%) | 1.0^a^ |
| Decreased range of motion | ***40 (41.7%)*** | 9 (22.5%) | 0.034* |
| Weakness | ***45 (46.9%)*** | ***19 (47.5%)*** | 0.95 |
| Tremor | 11 (11.5%) | 4 (10.0%) | 1.0^a^ |
| Dystonia | 5 (5.2%) | 1 (2.5%) | 0.67^a^ |
| Hair changes | 15 (15.2%) | 0 (0.0%) | 0.006*^a^ |
| Nail changes | 21 (21.9%) | 3 (7.5%) | 0.051^a^ |
| Skin changes | 13 (13.5%) | 3 (7.5%) | 0.39^a^ |
|  | | | |
| Hyperalgesia | **71 (73.4%)** | 11 (27.5%) | <0.0001* |
| Allodynia | **68 (70.8%)** | ***20 (50.0%)*** | 0.021* |
| Temperature changes | ***32 (33.3%)*** | 4 (10.0%) | 0.005*^a^ |
| Color changes | ***42 (44.2%)*** | 3 (7.5%) | <0.0001* |
| Edema | 28 (29.2%) | 3 (7.5%) | 0.007*^a^ |
| Sweating changes | 4 (4.2%) | 0 (0.0%) | 0.32^a^ |
| Decreased range of motion | ***42 (43.8%)*** | 11 (27.5%) | 0.077 |
| Weakness | ***53 (55.2%)*** | ***15 (37.5%)*** | 0.060 |
| Tremor | 1 (1.0%) | 1 (2.5%) | 0.50 |
| Dystonia | 11 (11.5%) | 3 (7.5%) | 0.76 |
| Hair changes | 14 (14.6%) | 1 (0.0%) | 0.010*^a^ |
| Nail changes | 13 (13.5%) | 0 (0.0%) | 0.011*^a^ |
| Skin changes | 13 (13.5%) | 2 (5.0%) | 0.23^a^ |

*Statistically significant; a=fisher’s exact test (all others used Chi-Square); **Bold** = >60% present, ***Bold italics*** = 30 - 60% present; Symptoms are self-reported by the patient whereas signs are observed on clinical examination.
